# Bovine Herpesvirus Type 1 (BHV-1) U_L_49.5 Luminal Domain Residues 30 to 32 Are Critical for MHC-I Down-Regulation in Virus-Infected Cells

**DOI:** 10.1371/journal.pone.0025742

**Published:** 2011-10-26

**Authors:** Huiyong Wei, Ying Wang, Shafiqul I. Chowdhury

**Affiliations:** Department of Pathobiological Sciences, School of Veterinary Medicine, Louisiana State University, Baton Rouge, Louisiana, United States of America; Ghent University, Belgium

## Abstract

Bovine herpesvirus type 1 (BHV-1) U_L_49.5 inhibits transporter associated with antigen processing (TAP) and down-regulates cell-surface expression of major histocompatibility complex (MHC) class I molecules to promote immune evasion. We have constructed a BHV-1 U_L_49.5 cytoplasmic tail (CT) null and several U_L_49.5 luminal domain mutants in the backbone of wild-type BHV-1 or BHV-1 U_L_49.5 CT- null viruses and determined their relative TAP mediated peptide transport inhibition and MHC-1 down-regulation properties compared with BHV-1 wt. Based on our results, the U_L_49.5 luminal domain residues 30–32 and U_L_49.5 CT residues, together, promote efficient TAP inhibition and MHC-I down-regulation functions. In vitro, BHV-1 U_L_49.5 Δ30–32 CT-null virus growth property was similar to that of BHV-1 wt and like the wt U_L_49.5, the mutant U_L_49.5 was incorporated in the virion envelope and it formed a complex with gM in the infected cells.

## Introduction

Bovine herpesvirus-1 (BHV-1) is an important cattle pathogen responsible for a wide variety of clinical diseases, including conjunctivitis and upper respiratory tract infection known as infectious bovine rhinotracheitis (IBR), reproductive tract lesions and abortion in pregnant cows, and systemic infection in the newborn [1], [2], [3]. In addition, BHV-1 has been recognized as an important component of the bovine respiratory disease (BRD) complex [3], [4]. Normally, proteosomally processed viral proteins yield peptides that bind to TAP heterodimer, consisting of the subunits TAP1 and TAP2 [5], [6], [7]. Following viral peptide binding, the TAP1/TAP2 heterodimer undergoes conformational changes [5], [6], [7]. Subsequently, peptides are transported into the ER and loaded onto MHC-I molecules to form MHC/peptide complexes which are transported to and presented on the cell surface [8], [9]. However, in BHV-1-infected cells, U_L_49.5 (BHV-1 homolog of envelope glycoprotein gN) binds to TAP, interferes with its peptide transport function and also degrades the TAP [10], [11]. Consequently, BHV-1 interferes with the MHC class I antigen presentation pathway and during the initial phase of viral infection, escapes host cellular immune surveillance and elimination [11]–[15]. Modified live vaccines (MLV) against BHV-1 including genetically engineered gE-deleted marker vaccines are being used for vaccination against BHV-1. However, problems associated with BHV-1 infection in the vaccinated animals exist, especially in the feedlot. Since both the traditional and gE-deleted MLVs have wild-type U_L_49.5, these vaccines like the wild-type virus, are transiently immunosuppressive. Therefore, there is a need for further improvement of the current MLVs [16]–[19].

**Figure 1:**
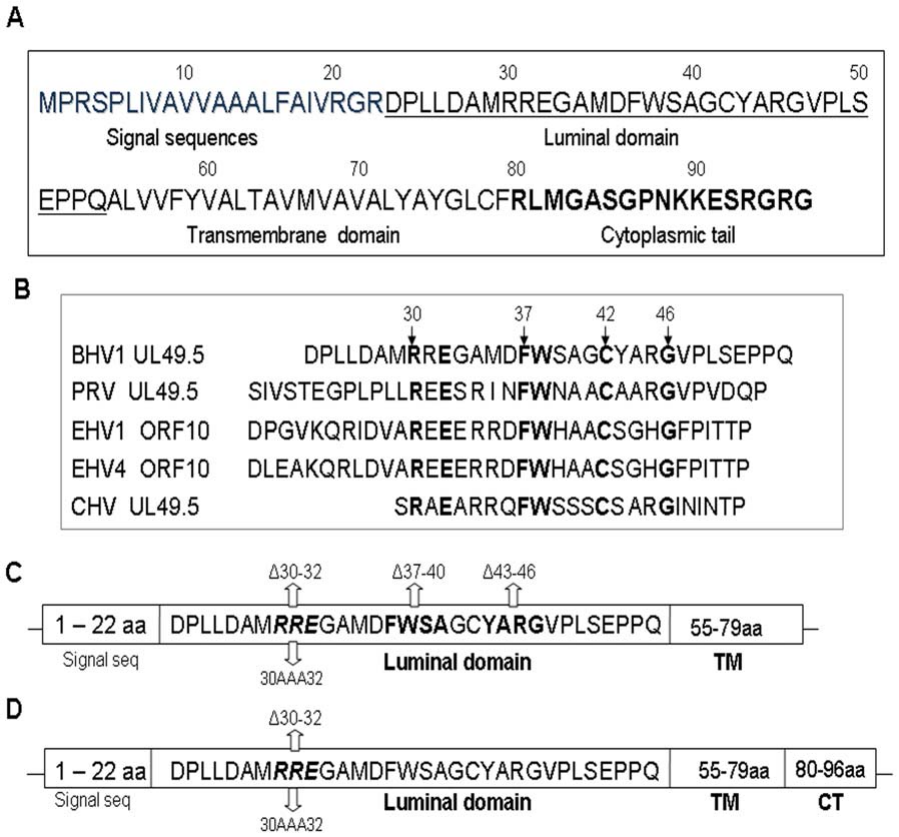
Predicted amino acid (aa) sequences of BHV-1 U_L_49.5 ORF. The bold italic letters indicate U_L_49.5 cytoplasmic tail residues (**A**). Alignment of BHV-1 U_L_49.5 luminal domain aa sequences with U_L_49.5 homologues of equine herpesvirus 1 (EHV-1; ref. or Genbank accession #), EHV-4, psueudorabiesvirus (PRV) and canine herpesvirus (CHV) (**B**). Identical aa residues are bolded. Schematic illustration of BHV-1 U_L_49.5 luminal domain mutants with a short sequence deletion or alanine substitutions using the BHV-1 U_L_49.5 CT-null (**C**) or BHV-1 wt as a backbone (**D**).

Alphaherpesvirus U_L_49.5 (gN) and gM homologs are associated with cellular and virion membranes and the gN homologs form complexes with envelope glycoprotein gM [20]–[22]. BHV-1 U_L_49.5 predicted ORF (Fig. 1A) is composed of an N-terminal signal sequence of 22 amino acids (aa), an extracellular luminal domain of 32 aa, a transmembrane (TM) domain of 25 aa, and a short cytoplasmic tail (CT) of 17 aa [10], [22], [23]. Previous results have shown that a BHV-1 U_L_49.5 CT-truncated virus lacking the cytosolic 17 amino acids no longer degraded bovine TAP molecules yet retained the TAP inhibition and MHC-I down-regulation functions [15]. This suggests that the BHV-1 U_L_49.5 luminal domain or TM domain is associated with the TAP inhibition function.

Recently, using N-terminal and C-terminal truncated versions of BHV-1 U_L_49.5 expressed in a Baculovirus expression vector system, it was shown that U_L_49.5 luminal domain residues 23–32 and U_L_49.5 cytoplasmic tail residues 94–96 are both essential for U_L_49.5-mediated degradation of human TAP [24]. However, U_L_49.5 luminal domain residues 28–32 alone are sufficient for human TAP inhibition and down-regulation of human MHC-I surface expression [24]. The goal for this study was to i) identify the exact U_L_49.5 residues critical for bovine TAP inhibition and bovine MHC-I down-regulation in the context of BHV-1 virus-infected Madin-Darby bovine kidney (MDBK) cells, and ii) determine the effect of U_L_49.5 mutations on U_L_49.5/gM complex formation and gM processing. We constructed BHV-1 U_L_49.5 luminal domain mutants, with a short sequence deletion, using a BHV-1 U_L_49.5 cytoplasmic tail (CT) null virus or wt BHV-1 as a backbone and analyzed their TAP inhibition and MHC-I molecule surface expression properties in infected MDBK cells relative to wt BHV-1. The results demonstrate that U_L_49.5 residues 30 to 32 are essential for the BHV-1 U_L_49.5-mediated TAP inhibition/MHC-I down-regulation function. However, U_L_49.5 CT residues are required for efficient TAP inhibition/MHC-I down-regulation. The mutant U_L_49.5 lacking luminal domain residues 30–32 and the entire cytoplasmic tail is incorporated in the virion envelope and it formed a complex with gM in the infected cells.

## Materials and Methods

### Cells and virus strain

The MDBK cell line was maintained in Dulbecco's modified Eagle's medium (DMEM) supplemented with 5–10% heat-inactivated fetal bovine serum (FBS). The BHV-1 Cooper (Colorado-1) strain, obtained from the American Type Culture Collection (Manassas, VA) was propagated and titrated in MDBK cells as previously described [25].

### Plasmids and bacterial strains

Vector pGEX-4T-2 (GE healthcare) and *E. coli* strain BL21 were used to express BHV-1 U_L_49.5-GST or bovine TAP1-GST fusion proteins. *E. coli* strain DH10B was used to maintain all the infectious BHV-1 BAC clones. *E. coli* strain SW105 (kindly provided by Dr. Copeland) was used for Red recombination.

### Antibodies

Chicken anti-Calreticulin (ER) polyclonal Ab (ab14234, Abcam), biotinylated donkey anti-rabbit IgG (ab6801, Abcam), HRP-conjugated donkey anti-rabbit IgG (Cat. 31458, Thermo), phycoerytrin (PE)-conjugated donkey anti-goat antibody (F0107, R&D), Alexa Flour (350)-conjugated donkey anti-chicken Ab (Invitrogen), Alexa flour 488-conjugated donkey anti-goat Ab (Molecular Probes), Alexa flour 594-conjugated donkey anti-rabbit Ab (Molecular Probes), mouse anti-MHC I Ab (H58A, VMRD), and FITC-conjugated rat anti-mouse IgG (ebioscience) were purchased from the respective commercial sources.

### Production of anti-BHV-1 U_L_49.5-, gM- and anti-bovine TAP1-specific polyclonal sera

#### i) Anti-BHV-1 gM-specific antibody

A peptide corresponding to the predicted amino acid (aa) residues 191–205 ([H]-QAVHALRERSPRAHRC-OH) of BHV-1 gM was synthesized and conjugated to polyethylene glycol as described earlier [26] [27] and used to immunize New Zealand white rabbits (Cocalico Biologicals) to generate anti-BHV-1 gM serum.

#### ii) Anti-BHV-1 U_L_49.5-specific antibody

The DNA fragment corresponding to U_L_49.5 amino acid residues 23 to 60 (Genbank Accession No. AJ004801) was cloned into pGEX-4T-2 vector and expressed in *E. coli* BL21 as a GST fusion protein. The U_L_49.5-specific peptide was purified using a glutathione-sepharose column followed by thrombin protease cleavage and used to immunize rabbits (Cocalico Biologicals) as described earlier [27].

#### iii) Anti-bovine TAP1-specific antibody

The predicted amino acid residues 117 to 167 and residues 351 to 415 (Accession No. AAY34698) of bovine TAP1 were amplified by PCR, cloned into Vector pGEX-4T-2, expressed as GST fusion proteins, and purified to immunize rabbits and goats as described above.

### Cell transfection and generation of U_L_49.5 expressing cell line

To generate a U_L_49.5 expressing cell line, first the entire BHV-1 U_L_49.5 ORF coding region was amplified from the wild type BHV-1 Cooper strain genomic DNA by PCR and cloned into the eukaryotic expression vector, pEF6/V5-His TOPO (Invitrogen). Positive clones containing U_L_49.5-specific sequences were identified by PCR and sequencing of the U_L_49.5 ORF coding region. One positive U_L_49.5-pEF6/V5-His clone DNA was transfected into MDBK cells by Lipofectamine (Invitrogen) as described earlier [27]. Forty-eight hours after transfection, confluent cells were treated with trypsin and diluted 5-fold in DMEM containing 10 µg/ml of Blastcidin (Invitrogen) and plated in 25 cm^2^ flasks. The Blastcidin concentration was decreased to 5 µg/ml after 7 days, and thereafter the medium was replaced every 3 days until the distinct Blasticidin-resistant colonies developed. Blastcidin-resistant clones were then isolated and analyzed for U_L_49.5 expression by indirect immunofluorescence (IF) and immunoprecipitation assays (Fig. S2).

### Radiolabelling of mock- or virus-infected MDBK cell proteins, SDS-PAGE and immunoprecipitation/immunoblotting analysis

[^35^S] methionine-cysteine labeling of the mock- or virus-infected MDBK cells was performed as described earlier [28], [29]. Cell lysates and immunoprecipitates were denatured at 100°C in reducing sample buffer containing β-mercapthoethanol for the U_L_49.5 samples, and separated in a 5∼20% linear gradient sodium dodecyl sulfate-polyacrylamide gel (SDS-PAGE) [23]. However, for gM and TAP1 analyses, cell lysates and immunoprecipitates were incubated at 56°C in reducing sample buffer with 100 mM Dithiothreitol (DTT) and then loaded on a 5∼20% linear gradient or 10% SDS-PAGE respectively [22], [23], [28].

### Construction of BHV-1 U_L_49.5 cytoplasmic tail null virus (BHV-1 U_L_49.5 CT-null BAC)

We followed a two-step Red-mediated mutagenesis protocol as described earlier by Tischer et al [30] to construct a U_L_49.5 cytoplasmic tail null virus. Briefly, primers for U_L_49.5-CT null-forward (for) and U_L_49.5-CT null-reverse (rev) were designed to delete 7 nucleotides (nt) of U_L_49.5 ORF (238 to 244, AGGCTCA) and to mutate the nt 246–247 (GG) to AA (Fig. S1, table 1). The PCR amplified I-SceI/aphAI cassette was digested with Dpn I, purified and electroporated together with pBAD-I-SceI DNA into SW105 competent cells harboring pBHV-1 WT BAC for 1^st^ recombination as described earlier [30], [31], [32]. Several kanamycin-sensitive colonies were obtained after the 2^nd^ recombination. Finally, positive pBHV-1 BAC U_L_49.5 CT-null colonies were identified by PCR and sequencing of the U_L_49.5 ORF in the putative mutant colonies. The BAC-excised BHV-1 U_L_49.5 CT-null virus was generated from a pBHV-1 BAC U_L_49.5 CT-null clone by Cre-mediated BAC excision as described earlier [30]–[32].

**Table 1:** PCR primers used for generation of BHV-1 U_L_ 49.5 mutants and for colony identification.

Primer	Sequence (5′ to 3′)		
**Mutagenesis** a			
**CT-null for**	5′-AATGGTCGCCGTGGCCCTGT*ACGCGTACGGGCTTTGCTTTTAAGCGCCAGCGGGCCCAAT*aggatgacgacgataagtaggg-3′		
**CT-null Rev**	5′-CGCCCCCGCGACTCCTTTTT*ATTGGGCCCGCTGGCGCTTAAAAGCAAAGCCCGTACGCGT*caaccaattaaccaattctgattag-3′		
**Δ30–32 for**	5′-TGCCATCGTGCGCGGCCGCG*ACCCCCTGCTAGACGCGATGGGGGCAATGGACTTTTGGAG*aggatgacgacgataagtaggg-3′		
**Δ30–32 rev**	5′-CGCGCGCGTAGCAGCCTGCG*CTCCAAAAGTCCATTGCCCCCATCGCGTCTAGCAGGGGGT*caaccaattaaccaattctgattag-3′		
**Δ37–40 for**	5′-ACCCCCTGCTAGACGCGATGC*GGCGCGAGGGGGCAATGGACGGCTGCTACGCGCGCGGGGT*aggatgacgacgataagtaggg-3′		
**Δ37–40 rev**	5′-GCGGTGGCTCCGAGAGCGGC*ACCCCGCGCGCGTAGCAGCCGTCCATTGCCCCCTCGCGCC*caaccaattaaccaattctgattag-3′		
**Δ43–46 for**	5′-ATGCGGCGCGAGGGGGCAATGG*ACTTTTGGAGCGCAGGCTGCGTGCCGCTCTCGGAGCCACC*aggatgacgacgataagtaggg-3′		
**Δ43–46 rev**	5′-TAAAAAACAACCAGGGCCTGC*GGTGGCTCCGAGAGCGGCACGCAGCCTGCGCTCCAAAAGT*caaccaattaaccaattctgattag-3′		
**30aaa32 Fo**	5′-TGCCATCGTGCGCGGCCGCGACCCCCTGCTAGACGCGATGgcggccgcgGGGGCAATGGACTTTTGGAGaggatgacgacgataagtaggg-3′		
**30aaa32 Re**	5′-CGCGCGCGTAGCAGCCTGCG*CTCCAAAAGTCCATTGCCCC*cgcggccgc*CATCGCGTCTAGCAGGGGGT*caaccaattaaccaattctgattag-3′		
**Colony PCR** b			
**U_L_ 49.5 forward**	agagcgccagcgagtcgggctc	**d30–32 SRe**	agtccattgcccc**CTCGCGCCG**
**d37–40 SRe**	gcgcgcgtagcagcc**TGCGCT**	**d43–46 SRe**	ctccgagagcggcac**CCCGC**

a BHV-1 U_L_ 49.5-specific sequences are shown in uppercase letter; the italicized and italicized-underlined sequences are complementary to each other in reverse orientation. Nucleotides in lowercase indicate the pEPkan-S-specific sequences.

b Primers used for identification of BHV-1 U_L_ 49.5 mutants from selected kanamycin-sensitive colonies by PCR. The bold letters indicate the complementary sequences, in reverse orientation, corresponding to the deleted U_L_ 49.5 nucleotides.

### BAC mutagenesis to incorporate short deletions or alanine substitutions within UL49.5 luminal domain

As shown in Table 1, primer pairs specific for short sequence deletion at U_L_49.5 amino acid residues 30 to 32 (U_L_49.5Δ30–32), 37 to 40 (U_L_49.5Δ37–40), and 43 to 46 (U_L_49.5Δ43–46) or alanine substitutions at residues 30 to 32 (U_L_49.5 30AAA32) were synthesized. The PCR products were amplified, purified, and electroporated into the SW105 competent cells harboring pBHV-1 BAC (wt) or pBHV-1 BAC U_L_49.5 CT-null for 1^st^ recombination, and 2^nd^ recombination was performed as described above. Reconstituted BAC containing or BAC-excised mutant viruses were then generated as described earlier [30], [31], [32]. BAC-excised U_L_49.5 mutant viruses were plaque purified and designated as BHV-1U_L_49.5Δ30–32, U_L_49.5 30AAA32, UL49.5Δ30–32 CT-null, U_L_49.5 30AAA32 CT-null, U_L_49.5Δ37–40 CT-null, and U_L_49.5Δ43–46 CT-null, respectively.

### Viral growth kinetics and plaque size determination

One step growth curve assays were performed as described earlier [25], [33]. Average plaque size was calculated by measuring 50 randomly selected plaques for each mutant virus (under a microscope with a graduated ocular objective).

### TAP1 and U_L_49.5 intracellular staining and laser scanning confocal microscopy

MDBK cells grown on permanox chamber slides (Cole-Parmer) were infected with wt BHV-1 or BHV-1 U_L_49.5 mutant viruses. Cells were fixed at different times post-infection with freshly prepared 1% paraformaldehyde in PBS, permeabilized with FACS permeabilizing solution, and blocked with 2% IgG free bovine serum albumin. The cells were incubated (for 60 minutes) with a cocktail of goat anti-bovine TAP 1 (1∶800), rabbit anti-U_L_49.5 (1∶3,200) and chicken anti-calreticulin (ER) antibodies (1∶3,200), washed and subsequently stained (for 15 min) with a cocktail of Alexa flour 488-conjugated donkey anti-goat (1∶2,000), Alexa 350-conjugated donkey anti-chicken (1∶2,000), and Alexa flour 594-conjugated donkey anti-rabbit antibodies (1∶2,000). After 5 washes, coverslips were mounted onto slides and examined with a Zeiss LSM510 laser scanning confocal microscopy using 20× and 40× objectives. Cellular ER-, bovine TAP1- and U_L_49.5-specific labeling was excited at laser wavelengths of 410 nm, 517 nm, and 617 nm respectively.

### Analysis of peptide transport in the BHV-1 U_L_49.5 mutants-infected cells

MDBK cells grown on 6-well plate were infected at a MOI of 10 with BHV-1 wt, BHV-1 U_L_49.5 CT-null and various U_L_49.5 luminal domain mutant viruses. At different time-points (2, 5, and 8 hpi), the cells were trypsinized, permeabilized, and incubated with 5 µl of Phycoerythrin-conjugated mouse anti-calreticulin (anti-ER) MAb (Clone FMC 75, Abcam) and the FITC-conjugated synthetic peptide, SVNKTERAY, in the absence and presence of ATP (10 mM, Sigma) for 20 min at 37°C in the dark [11], [34]. The cells were fixed after three washes and analyzed for fluorescence intensity by a FACS Calibur flow cytometer. At least 30,000 gated events based on forward and side scatters and pulse width were analyzed using Summit Data Acquisition and Analysis software (DakoCytomation). The mock infected MDBK cells served as a control.

### Detection of MHC-I expression on the infected cell surface by FACS

Approx. 10^6^ of MDBK cells either mock infected or infected (1 MOI) with BHV-1 wt, BHV-1 U_L_49.5 CT-null, or individual BHV-1 mutant virus were collected at 12 or 18 hpi, blocked with IgG free BSA, and incubated (for 20 min) with mouse anti-bovine MHC I Ab. After PBS washes, cells were incubated with 5 µl of FITC-conjugated rat anti-mouse Ab and fixed with 2% formalin and then analyzed by flow cytometer. The stained cells were gated based on forward and side scatters and pulse width. At least 30,000 gated events were analyzed using Summit Data Acquisition and Analysis Software (DakoCytomation) as previously described [35]. MDBK cells infected similarly with the respective viruses were stained by FITC-conjugated mouse IgG2a and used as the isotype controls.

## Results

### Construction and characterization of BHV-1 UL49.5 CT-null virus and UL49.5 luminal domain mutants

We constructed a BHV-1 U_L_49.5 CT-null mutant virus by deletion of two predicted amino acid residues 80RL81 encoded by nt 238-AGGCTCA-244 (Fig. 1A and Fig. S1). In addition, nt 246GG247 were mutated to AA thereby incorporating a strong stop codon, TAA immediately downstream of the predicted aa residue 79F (Fig. 1A and Fig. S1).

Based on an alignment of predicted amino acid sequences of U_L_49.5 luminal domains of several animal alpha herpesviruses; BHV-1, pseudorabiesvirus (PRV, Accession No. U38547.1), equine herpesvirus 1 type (EHV-1, Accession No. AY665713.1), equine herpesvirus type 4 (EHV-4, Accession No. NC_001844), and canine herpesvirus (CHV, Patent EPO 910406), we noticed that a BHV-1 luminal domain motif U_L_49.5 30-RXEXXXXFW-XXXCXXXG-46 is conserved within the corresponding U_L_49.5 luminal domains of several alpha herpesviruses (Fig. 1B). To determine whether any of these conserved sequences are functionally important for TAP inhibition, we designed and constructed BHV-1 U_L_49.5 luminal domain mutants (U_L_49.5Δ30–32, U_L_49.5Δ37–40 and U_L_49.5Δ43–46) initially, in the BHV-1 U_L_49.5-CT null backbone (Fig. 1C). To determine the role of residues 30–32 alone in TAP inhibition and TAP degradation, we constructed both U_L_49.5Δ30–32 and alanine exchanged (U_L_49.5 30AAA32) mutants, later, in the BHV-1 wt backbone (Fig. 1D).

**Figure 2:**
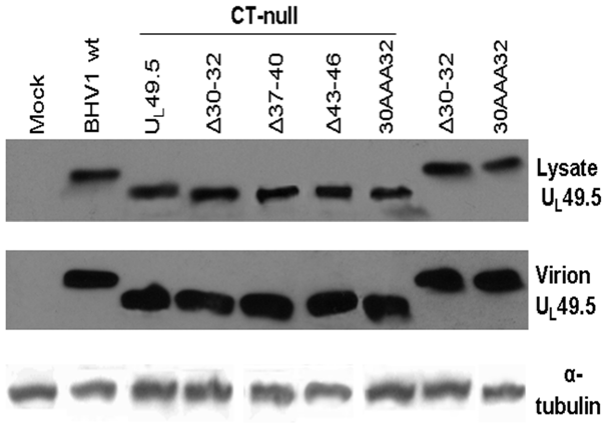
Immunoblotting analysis of mutant U_L_49.5 expressed in the infected cells and incorporated in the virion envelope by various U_L_49.5 mutant viruses. Uninfected (Mock), BHV-1 wt- or various mutant virus infected cell lysates or partially purified virions were separated by a 10–20% gradient SDS-PAGE and incubated with diluted (1∶400) rabbit anti-BHV-1 α- U_L_49.5 specific antibodies. Equal amounts of the extracted cell lysates or virions were loaded, and the α–tubulin was used as a control.

BAC excised BHV-1 U_L_49.5 mutant viruses were analyzed by immuno-precipitation and/or immunoblotting to determine molecular mass and level of mutant U_L_49.5 expression, incorporation of the mutant U_L_49.5 in the envelope and effect of U_L_49.5 mutation on gM processing or U_L_49.5/gM interaction. As shown in Figure 2A, relative to wt U_L_49.5 (approx. molecular mass 10 kDa), molecular mass of the CT-null U_L_49.5 is approx. 8 kDa, which is consistent with the predicted molecular mass of U_L_49.5 lacking C-terminal 17 aa residues. BHV-1 U_L_49.5Δ30–32 CT-null, U_L_49.5Δ37–40 CT-null, U_L_49.5Δ43–46 CT-null, and U_L_49.5 30AAA32 CT-null viruses all have a U_L_49.5 with molecular mass of approx. 8 kDa which are similar to the parental virus BHV-1 U_L_49.5 CT-null. However, the BHV-1 U_L_49.5Δ30–32 and U_L_49.5 30AAA32 have an approx. molecular mass of 10 kDa similar to wt U_L_49.5 (Fig. 2). Importantly, all mutant U_L_49.5 proteins were incorporated into their respective virion envelopes (Fig. 2).

**Figure 3:**
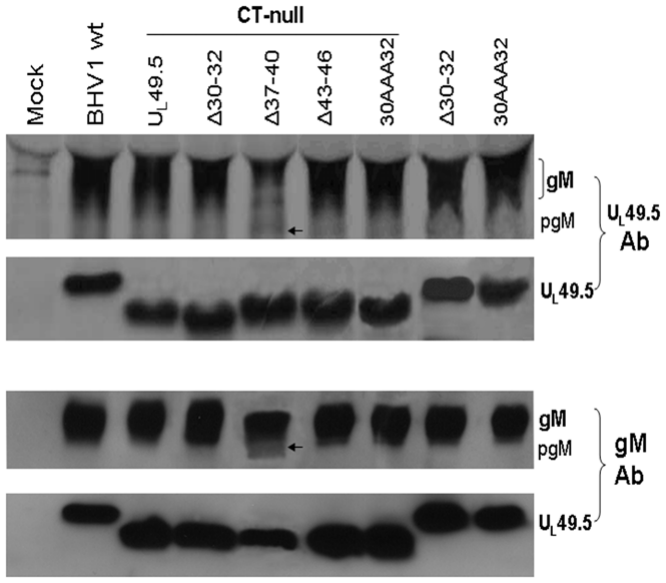
Analysis of gM-U_L_49.5 interaction by radioimmunoprecipitation assay (RIPA). ^35^S labeled lysates from the mock-infected or virus-infected MDBK cells were immunoprecipitated with undiluted rabbit anti U_L_49.5- (**A**) or anti gM-specific polyclonal antibodies (**B**) and separated by SDS-PAGE and visualized by autoradiography. Unprocessed gM (pgM, marked with an arrow) and processed gM (gM) are marked.

Results of immunoprecipitation using U_L_49.5- or gM-specific rabbit polyclonal antibodies showed that i) amounts of wt and mutant U_L_49.5 CT-null, U_L_49.5Δ30–32 CT-null, U_L_49.5Δ43–46 CT-null, and U_L_49.5Δ37–40 CT-null) immunoprecipitated by anti U_L_49.5 antibody in each case were very similar (Fig. 3), ii) the amount and molecular mass of mature gM coimmunoprecipitated by the anti U_L_49.5 antibody from infected cell lysates of BHV-1 wt, BHV-1 U_L_49.5 CT-null, BHV-1 U_L_49.5Δ30–32 CT-null, and BHV-1 U_L_49.5Δ43–46 CT-null viruses were very similar but in the case of BHV-1 U_L_49.5Δ37–40 CT-null, the amount of processed (mature) gM coimmunoprecipitated was reduced (Fig. 3), and iii) consistent with the latter results, with the exception of BHV-1 U_L_49.5Δ37–40 CT-null, anti gM antibody coimmunoprecipitated similar amounts of U_L_49.5 from the all the virus-infected cell lysates. In the case of BHV-1 U_L_49.5Δ37–40 CT-null, the amount of U_L_49.5 coimmuno-precipitated was slightly reduced and additionally, there was an increase in the amount of unprocessed gM (pgM) (Fig. 3).

Taken together, these results indicated that the mutant viruses have essentially normal U_L_49.5 expression and all but one mutant (U_L_49.5Δ37–40 CT-null) have a normal gM processing and U_L_49.5/gM interaction. The BHV-1 U_L_49.5Δ37–40 CT-null virus has slightly defective gM processing and U_L_49.5/gM interaction.

### Growth characteristics of reconstituted U_L_49.5 mutant viruses in vitro in MDBK cells

**Figure 4:**
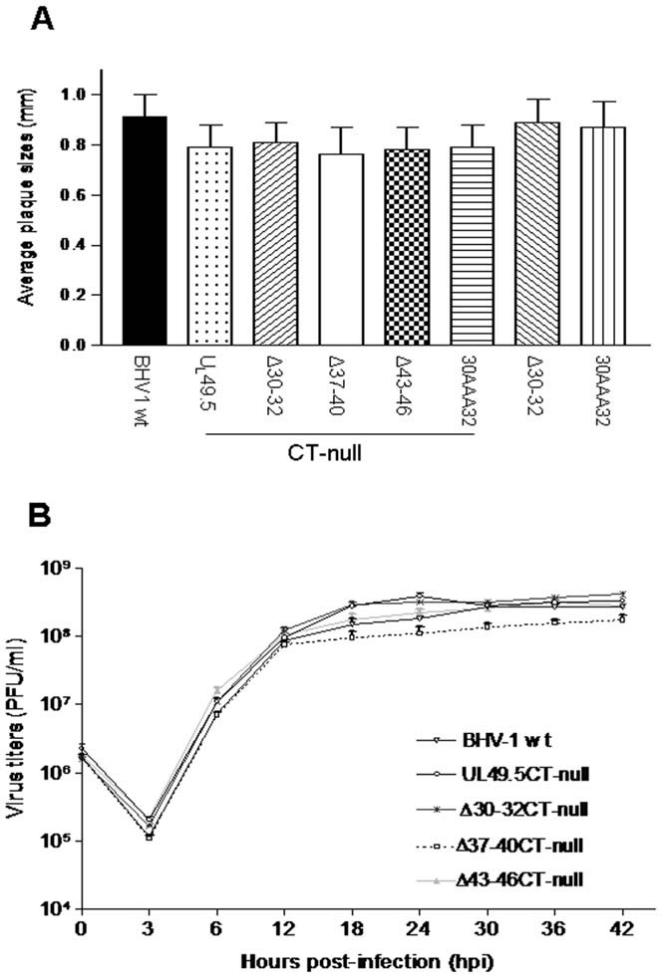
Plaque morphology and growth kinetics of BHV-1 U_L_49.5 mutants in MDBK cells. (**A**) Plaque sizes produced by BHV-1 wt, and various BHV-1 U_L_49.5 mutant viruses were measured at 48 hpi. Average plaque diameters of 50 randomly selected plaques are shown as mean ± standard deviation. (**B**) One-step growth kinetics of BHV-1 wt, three representative BHV-1 U_L_49.5 mutants (U_L_49.5 Δ30–32, U_L_49.5 Δ37–40 and U_L_49.5Δ43–46) in the CT-null backbone. Note that BHV-1 U_L_49.5Δ37–40 CT-null exhibited slightly delayed viral replication kinetics between 12 to 42 hpi. Each data point represents the average of duplicate samples obtained from separate infections.

The results of average plaque sizes (Figure 4A) and one step growth kinetics (Figure 4B) showed that all BHV-1 U_L_49.5 mutants had virtually similar plaque sizes and growth kinetics when compared with BHV-1 wt.

### Peptide transport in BHV-1 U_L_49.5 mutant virus-infected cells

**Figure 5:**
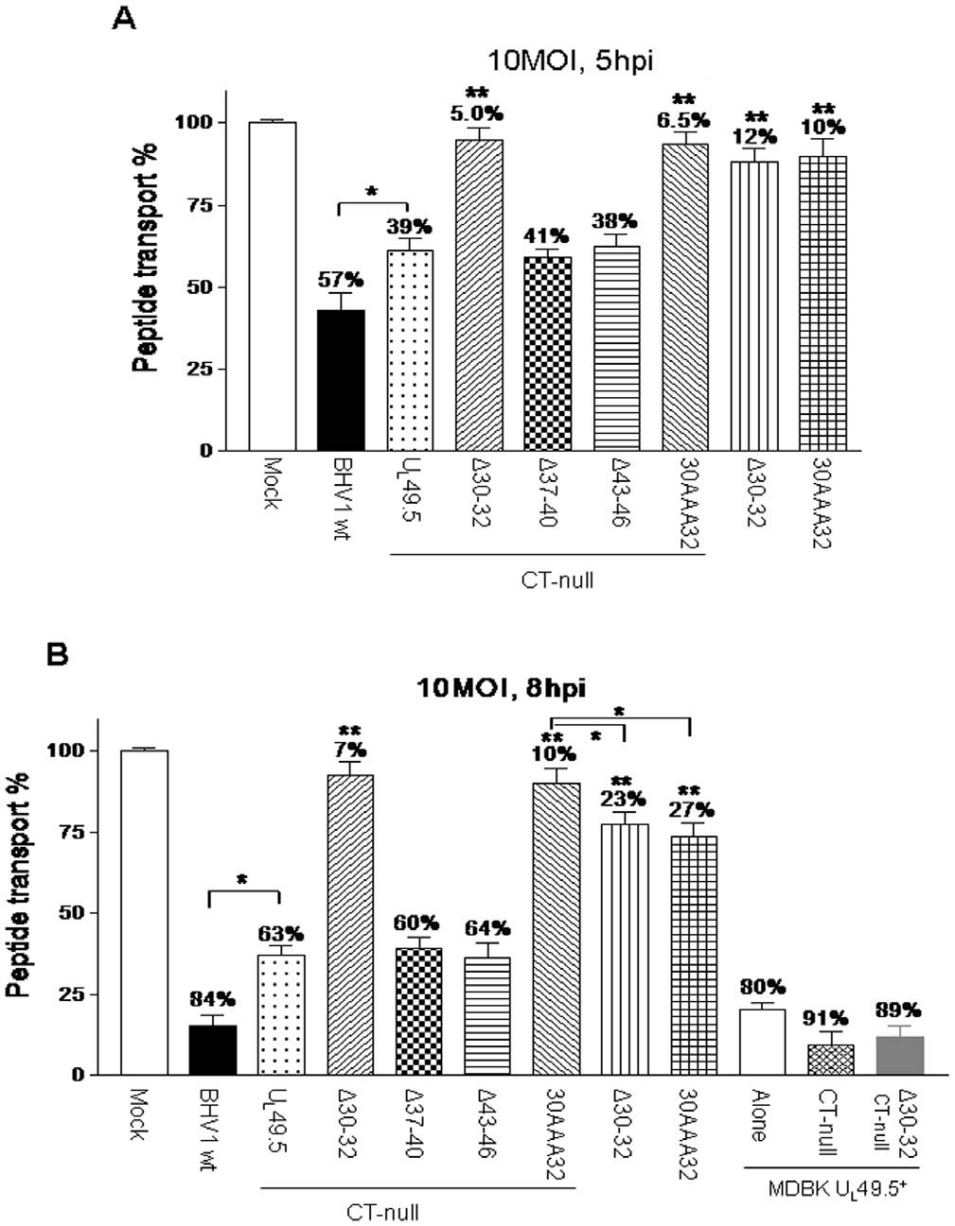
Analysis of TAP peptide transport function in various BHV-1 U_L_49.5 mutant-infected cells. The mock-, BHV-1 wt or various BHV-1 U_L_49.5 mutant virus-infected MDBK cells (10 MOI) were harvested at 5 hpi (A) and 8 hpi (B) to perform peptide transport assays as described in the materials and methods. The percentage above the bars denotes the decreased peptide-specific FITC intensity for each sample when compared with the mock-infected cells, and the statistical significance is indicated by stars: *, P<0.05; **, P<0.01.

Peptide transport was assessed, in the presence (Fig. 5A and B) and absence of ATP (data not shown), in mock-infected MDBK cells and MDBK-U_L_49.5^+^ cells or infected with BHV-1 wt, BHV-1 U_L_49.5 CT-null and individual BHV-1 U_L_49.5 mutant viruses. At 5hpi the effect of U_L_49.5 mutations on peptide transport in BHV-1 U_L_49.5 mutant virus-infected cells relative to BHV-1 wt-infected cells was noticeable (Fig. 5A), but the effects were more prominent at 8 hpi (Fig. 5B). Relative to peptide transport in BHV-1 wt-infected MDBK cells at 8hpi, peptide transport in cells infected with: i) BHV-1 U_L_49.5Δ30–32 and BHV-1 U_L_49.5 30AAA32 was increased 4 fold (23% and 27% inhibition, respectively versus 84% inhibition for the wt; P<0.01) ii) BHV-1 U_L_49.5 CT-null was increased 2.5 fold (63% inhibition versus 84% for the wt; P<0.05), and iii) BHV-1 U_L_49.5Δ30–32 CT-null or BHV-1 U_L_49.5 30AAA32 CT-null was increased to approx. 6 folds (7% and 10% inhibition, respectively versus 84% for the wt; P<0.01) (Fig. 5B). Since there was also an additional 1.2–1.5 fold increase of peptide transport in BHV-1 U_L_49.5Δ30–32 CT-null-/BHV-1 U_L_49.5 30AAA32 CT-null-infected cells compared with the corresponding BHV-1 U_L_49.5Δ30–32/BHV-1 U_L_49.5 30AAA32 cells (7%/10% versus 23%/27% inhibition, respectively)(Fig. 5), the increase in peptide transport due to the U_L_49.5 CT sequence deletion alone was also significant (P<0.05). As expected, in the absence of ATP there was no peptide transport in wt and mutant viruses-infected MDBK cells (data not shown).

Regardless of BHV-1 U_L_49.5 Δ30–32 or BHV-1 U_L_49.5Δ30–32 CT-null or BHV-1 U_L_49.5 CT-null virus infection, at 8hpi, peptide transport in infected MDBK U_L_49.5^+^ cells (Fig. S2) was inhibited to almost wt U_L_49.5 level (∼90%) (Fig. 5B). Therefore, the relative increase in peptide transport in MDBK cells infected with BHV-1 U_L_49.5 mutants with residues 30–32-deleted or substituted with alanine and BHV-1 U_L_49.5 CT-null virus is due to the lack of U_L_49.5 residues important for TAP mediated peptide transport function.

### MHC class I cell-surface expression in BHV-1 U_L_49.5 mutants-infected cells

**Figure 6:**
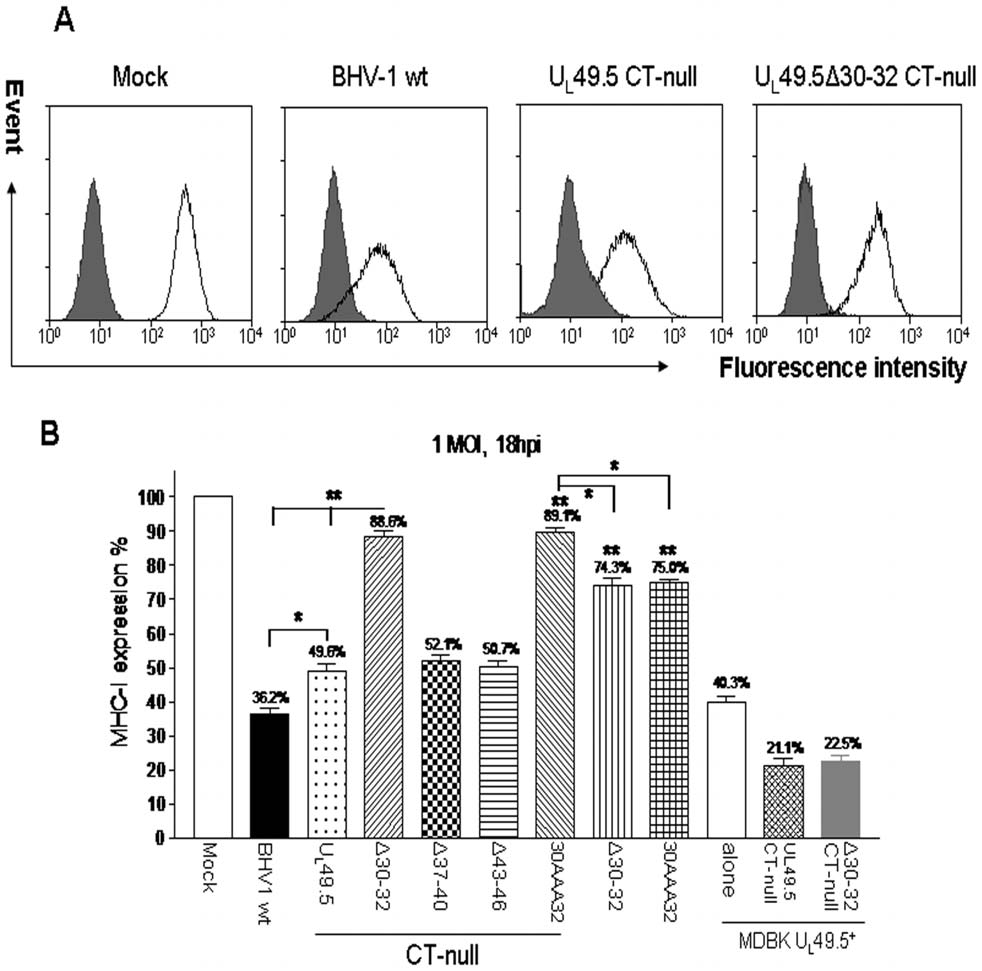
FACS analysis of MHC-I surface expression on various BHV-1 U_L_49.5 mutant-infected cells. Infected MDBK cells or U_L_49.5 expressing MDBK-U_L_49.5+ cells were stained with anti-MHC-I Ab, subsequently incubated with FITC-conjugated rat anti-mouse IgG and subjected to FACS analysis. **A**. Representative FACS histograms show the amount/profile of MHC-I expression in the mock-, BHV-1 wt-, BHV-1 U_L_49.5 CT-null- and BHV-1 U_L_49.5Δ30–32 CT-null-infected cell surface. Normal mouse IgG2a served as an isotype-matched control (filled curve). **B.** Results shown are the mean of three independent experiments of MHC-I expression on the MDBK cells or MDBK-U_L_49.5+ cells infected with BHV-1 wt, or various BHV-1 U_L_49.5 mutant viruses. The statistical significance is indicated by stars: *, P<0.05; **, P<0.01.

As shown in Fig. 6A, compared with MHC-I cell surface expression in mock-infected MDBK cells, there was reduced MHC-1 expression in cells infected with BHV-1 wt and BHV-1 U_L_49.5 CT-null viruses but not when cells were infected with BHV-1 U_L_49.5 Δ30–32 CT-null virus (Fig. 6A). As shown in Figure 6B, compared with BHV-1 wt-infected MDBK cells, MHC-I surface expression in: i) BHV-1 U_L_49.5Δ30–32- and BHV-1 U_L_49.5 30AAA32-infected MDBK cells was increased 2 fold (74.3% and 75.0%, respectively versus 36.2% for the wt; P<0.01) ii) BHV-1 U_L_49.5 Δ30–32 CT-null- and BHV-1 U_L_49.5 30AAA32 CT-null-infected MDBK cells was increased 2.5 fold (88.6% and 89.1%, respectively versus 36.2% for the wt; P<0.01) and iii) BHV-1 U_L_49.5 CT-null-infected cells was increased by 1.3 fold (49.6% versus 36.2% for the wt; P<0.05). Since a similar 1.2–1.3 fold increase in MHC-I surface expression was also obtained in BHV-1 U_L_49.5Δ30–32 CT-null-/BHV-1 U_L_49.5 30AAA32 CT-null- compared with BHV-1 U_L_49.5Δ30–32/BHV-1 U_L_49.5 30AAA32 –infected cells (88.6%/89% versus 74.3%/75%), the increase in MHC-I expression due to the deletion of U_L_49.5 CT sequences alone was also significant (P<0.05).

In MDBK U_L_49.5^+^ cells, regardless of BHV-1 U_L_49.5 CT-null or BHV-1 U_L_49.5Δ30–32 CT-null virus infection, MHC-I cell-surface expression was down-regulated like in wt BHV-1-infected MDBK cells (Fig. 6B). Therefore, the relative increase in MHC-I surface expression observed for the mutant viruses is due to the effect of specific U_L_49.5 mutation(s) and is consistent with the peptide transport data obtained for respective mutants with wt or U_L_49.5 CT-null backgrounds.

Taken together, even though U_L_49.5 residues 30–32 are essential for TAP mediated peptide transport/MHC-I down-regulation, U_L_49.5 CT residues are important to express full potential of U_L_49.5 mediated TAP inhibition and MHC-I down-regulation function.

### Status of TAP 1 molecules in the BHV-1 wt versus BHV-1 U_L_49.5 CT-null, BHV-1 U_L_49.5Δ30–32CT-null, BHV-1U_L_49.5Δ30–32 and BHV-1 U_L_49.5 30-AAA32 mutants-infected cells

**Figure 7:**
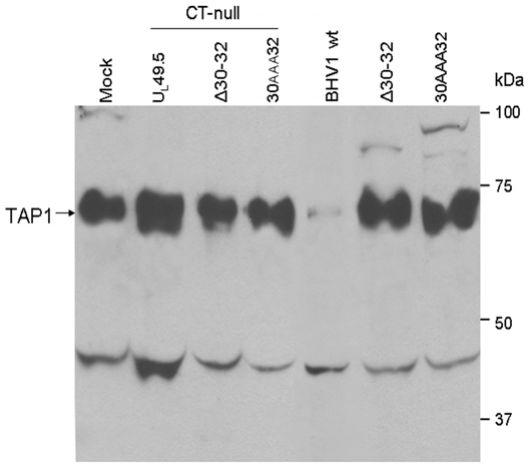
Immunoprecipitation/immunoblotting analysis of cellular TAP1. Mock- or various virus-infected cell lysates were immunoprecipitated with undiluted goat anti-bovine TAP1 specific antibody followed by immunoblotting with diluted (1∶200) rabbit anti-TAP1 polyclonal serum, and then developed by enhanced chemiluminescence. A ∼70 kDa TAP1 specific band is detectable in the mock-, BHV-1 U_L_49.5 CT-null-, BHV-1 U_L_49.5Δ30–32 CT-null, BHV-1 U_L_49.5 30AAA32 CT-null-, BHV-1 U_L_49.5Δ30–32-, and BHV-1 U_L_49.5 30AAA32-infected cell lysates, but not in the case of BHV-1 wt. Note that in case of BHV-1 wt virus-infected lysate TAP1-specific ∼70 kDa band is mostly degraded. A 45 kDa (approx.) non-specific protein is also immunoprecipitated and recognized in the immunoblot by the TAP1-specifc antibodies in all the samples including the BHV-1 wt. Relative amount of 45 kDa protein immunopreciptated from each cell lysate sample can be viewed as loading control.

Previously, we reported that in BHV-1 U_L_49.5 CT-null virus-infected cells, TAP1 is not degraded [24]. In this study, the effect of U_L_49.5Δ30–32, U_L_49.5Δ30–32 CT-null, U_L_49.5 30AAA32 and U_L_49.5 30AAA32 CT-null mutations on the status of TAP1 in the respective mutant virus-infected cells was determined and compared with the effect of U_L_49.5 CT-null and wt U_L_49.5 on TAP1 degradation (Fig. 7). Since TAP1 was degraded only in BHV-1 wt infected MDBK cells but not in BHV-1 U_L_49.5 CT-null-, BHV-1 U_L_49.5Δ30–32, BHV-1 U_L_49.5 30AAA32, BHV-1 U_L_49.5Δ30–32 CT-null and BHV-1 U_L_49.5 30AAA32 CT-null-infected MDBK cells (Fig. 7), both U_L_49.5 residues 30–32 and U_L_49.5 cytoplasmic tail sequences are equally important for U_L_49.5-mediated TAP degradation.

### Sub-cellular localization of wt and mutant U_L_49.5 in virus-infected cells

**Figure 8:**
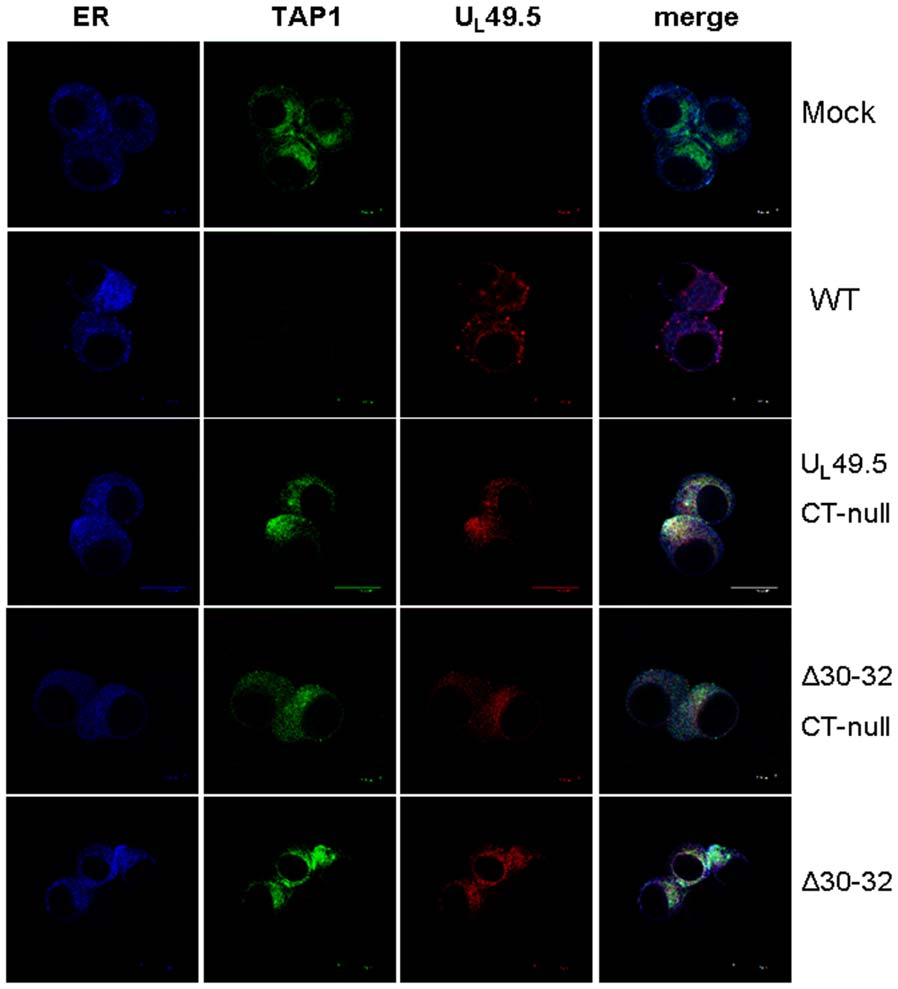
Analysis of TAP1 and mutant U_L_49.5 co-localization in the ER of BHV-1 wt- and BHV-1 U_L_49.5 mutant virus-infected cells. Mock-, BHV-1 wt (WT), BHV-1 U_L_49.5 CT-null, BHV-1 U_L_49.5Δ30–32 CT-null and BHV-1 U_L_49.5Δ30–32-infected MDBK cells were fixed at 12 hpi, permeabilized, and incubated with the cocktail of antibodies (goat anti-bovine TAP1, rabbit anti U_L_49.5, and chicken anti-Calreticulin/ER-specific). After washing, the samples were further incubated with cocktail of secondary antibodies (Alexa Flour 488-conjugated donkey anti goat, Alexa 350-conjugated donkey anti-chicken and Alexa flour 594-conjugated donkey anti rabbit). Note that TAP was not detectable in the ER of BHV-1 wt-infected cells. Mock-infected MDBK cells served as controls.

To determine the sub cellular localization of the mutant U_L_49.5 CT-null, U_L_49.5Δ30–32 CT-null and U_L_49.5Δ30–32 proteins relative to wt U_L_49.5 in the virus-infected cells and to determine the status of TAP 1 degradation and TAP1 co-localization with the mutant U_L_49.5, confocal microscopy was performed. The results showed that at 12 hpi, TAP1 was not detectable in wt-infected cells but TAP1 molecules were detectable in the ER of U_L_49.5 CT-null- , U_L_49.5Δ 30–32 CT-null- and U_L_49.5Δ30–32- infected cells (Fig. 8). Nevertheless, at 6 hpi minor amounts of TAP1 were detectable in the ER of BHV-1 wt-infected MDBK cells which colocalized with U_L_49.5 (data not shown).

Considering the TAP1 degradation, peptide transport, MHC-I down-regulation, and U_L_49.5-TAP1 co-localization data, we can conclude that TAP1 and U_L_49.5 residues 30–32 interaction in the ER is sufficient to inhibit TAP mediated peptide transport function. However, both U_L_49.5 residues 30–32 and the U_L_49.5 CT residues are required for maximum U_L_49.5 inhibition of TAP function.

## Discussion

Previous studies reported that following BHV-1 infection, TAP function was inhibited and as a consequence MHC-I surface expression was down-regulated [10], [11]. Our results from a recent study using a cytoplasmic tail truncated BHV-1 U_L_49.5Am80 virus confirmed this finding and further demonstrated that BHV-1 U_L_49.5Am80 did not degrade TAP molecules but retained TAP inhibition/MHC-I down-regulation functions in infected MDBK cells [15]. Therefore, the results implied that the functional domain required for TAP inhibition/MHC-I down-regulation maps elsewhere within U_L_49.5. We then constructed two BHV-1 U_L_49.5 transmembrane domain-deleted mutants (BHV-1 U_L_49.5Δ55–66 and BHV-1 U_L_49.5Δ 67–79) and determined that in both cases mutant U_L_49.5 protein affected gM processing, and that both viruses had defective replication properties (data not shown). Additionally, we and others (Wiertz, personal communication) found that the mutant U_L_49.5 versions lacking the transmembrane domain residues were not stable and were degraded (data not shown).

In this study, we constructed BHV-1 U_L_49.5 luminal domain mutants in the wt U_L_49.5 or U_L_49.5 CT-null backgrounds and determined their TAP inhibition, TAP degradation and MHC-I down-regulation properties in virus-infected MDBK cells relative to mock- and BHV-1 wt-infected MDBK cells. Further, we determined the effects of U_L_49.5 mutations that abolished the U_L_49.5-mediated TAP inhibition/MHC-I down-regulation functions (BHV-1 U_L_49.5Δ 30–32 CT-null) on virus replication and U_L_49.5/gM interaction. The salient features of our analyses were as follows: i) Relative to wt-infected cells, cells-infected with U_L_49.5 mutants having residues 30–32 deleted or substituted with alanine have significantly increased TAP mediated peptide transport and MHC-I surface expression. However, when BHV-1 U_L_49.5Δ30–32 mutation is combined with deletion of the U_L_49.5 CT residues, there was a small but significant additional increase in peptide transport and MHC-I surface expression, ii) deletion of U_L_49.5 residues 30–32 or U_L_49.5 CT-null mutation individually prevented U_L_49.5-mediated bovine TAP 1 degradation iii) BHV-1 U_L_49.5Δ 30–32 mutant virus replicated with wild-type efficiency in MDBK cells and iv) U_L_49.5Δ30–32/gM interaction and gM processing in mutant virus-infected cells were not affected.

Recently, using Baculovirus and/or transiently expressed mutant U_L_49.5 proteins, it was reported that deletion of U_L_49.5 luminal domain residues 23–32 or U_L_49.5 cytoplasmic tail residues 94–96 can prevent U_L_49.5-mediated human TAP degradation. However, U_L_49.5 luminal domain residues 28–32 alone are necessary for U_L_49.5 mediated human TAP inhibition and down-regulation of human MHC-I surface expression [24]. Based on our sequence alignments of the U_L_49.5 luminal domains of *Varicelloviruses*, including BHV-1, PRV, EHV-1 and EHV-4, we noticed a conserved motif U_L_49.5 30-RXEXXXXFWXXXCXXXG-46. Therefore, we questioned their functional relevance, *in vitro*, in the context of BHV-1-infected MDBK cells relative to the results obtained above with Baculovirus expressed U_L_49.5. Initially, we constructed several U_L_49.5 luminal domain mutants in which either residues 30–32 (RRE) or 37–40 (FWSA) or 43–46 (YARG) were deleted in the background of a U_L_49.5 CT-null virus and analyzed their effect on gM processing and interaction with gM. Most importantly, their TAP inhibition/MHC-I down-regulation properties were analyzed in comparison to wt U_L_49.5 and U_L_49.5 CT-null.

The results indicated that while increases in TAP-mediated peptide transport and MHC-I cell-surface expression in BHV-1 U_L_49.5 CT-null infected MDBK cells were significant when compared with BHV-1 wt, additional deletion of BHV-1 U_L_49.5 residues 30–32 (RRE) resulted in even further significant increases in peptide transport and MHC-I cell surface expression when compared with BHV-1 wt and BHV-1 U_L_49.5 CT-null. Subsequently, we introduced the U_L_49.5Δ30–32 deletion or U_L_49.5 30AAA32 substitutions in BHV-1 wt and determined their TAP inhibition/MHC-I down-regulation and TAP degradation functions in comparison to wt U_L_49.5 and U_L_49.5 CT-null. Based on the results, deletion or alanine exchange of U_L_49.5 residues 30–32 or U_L_49.5 lacking the CT residues alone prevented U_L_49.5-mediated bovine TAP 1 degradation. Relative to wt U_L_49.5 or U_L_49.5 CT-null, there was a significant increase in peptide transport and MHC-I cell surface expression in U_L_49.5 30–32 alanine substitution or deletion mutations in the wt background. Nevertheless, peptide transport and MHC-I surface expression was increased even more when U_L_49.5 Δ30–32 and U_L_49.5 30AAA32 mutations were introduced in the U_L_49.5-CT null background. When MDBK cells expressing the wt U_L_49.5 were infected with the BHV-1 U_L_49.5-CT null-, BHV-1 U_L_49.5Δ30–32 and BHV-1 U_L_49.5 30AAA32 mutant viruses, the inhibition of peptide transport and down- regulation of MHC-I cell surface expression of the mutant viruses were restored to the BHV-1 wt levels. Taken together, the results of TAP1 degradation, peptide transport inhibitory and MHC-I down-regulatory property of the BHV-1 U_L_49.5 CT-null, BHV-1 U_L_49.5Δ30–32, BHV-1 U_L_49.5 30AAA32 mutants in MDBK cells and MDBK cells expressing wt U_L_49.5, we conclude that i) U_L_49.5 residues 30–32 (RRE) and U_L_49.5 CT residues (80–96) both are important for TAP1 degradation, ii) even though primary TAP inhibition domain lies in U_L_49.5 30–32 residues, the fullest extent of U_L_49.5 mediated TAP inhibition function additionally required the U_L_49.5 cytoplasmic tail (CT) residues. Similarly, the fullest extent of U_L_49.5-mediated down-regulation of MHC-I cell surface expression required both the U_L_49.5 luminal domain residues 30–32 and the U_L_49.5 CT residues.

Our results, in general, are in agreement with the results of Baculovirus expressed U_L_49.5 in a transient expression system, especially with respect to U_L_49.5 sequences important for TAP degradation [24]. However, our results differed with respect to TAP inhibition and MHC-I surface expression [24]. Our TAP inhibition results showed that even though U_L_49.5 residues 30–32 are essential for TAP inhibition/MHC-I down-regulation functions, the fullest extent of U_L_49.5 mediated TAP inhibition and MHC-I down-regulation additionally required the U_L_49.5 cytoplasmic tail (CT) residues. In summary, the U_L_49.5 residues 30–32 and U_L_49.5 CT residues together inhibit peptide transport and down- regulate MHC-I cell surface expression.

**Figure 9:**
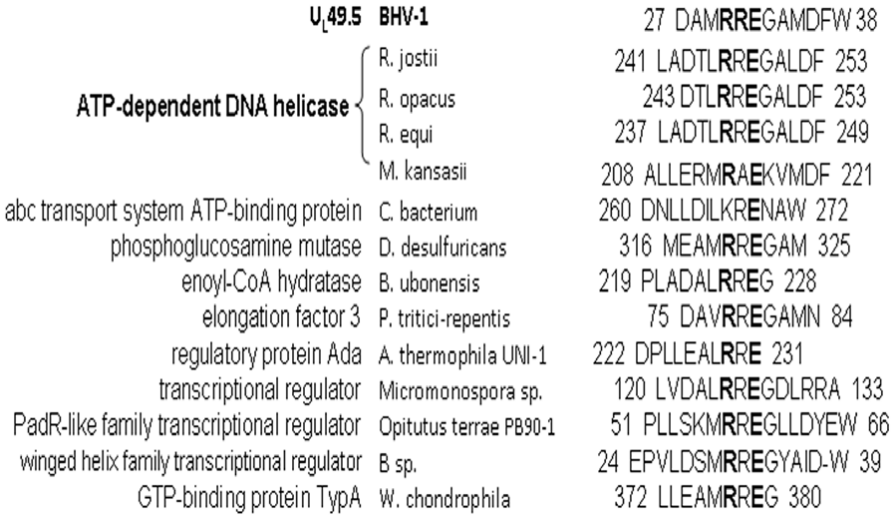
Alignment of the RXE consensus motif obtained from a BLAST search of the NCBI protein data bank. GenBank accession numbers are listed within parenthesis. ATP-dependent DNA helicases in *R. jostii* (ABG98153.1), *R. opacus* (BAH54685.1), *M. kansasii* (ZP_04750796.1) and *R. equi* (CBH49273.1). ATP-binding protein of abc transport system in *C. bacterium* (EDZ61139.1). Phosphoglucosamine mutase in *D. desulfuricans subsp* (ACL49611.1). enoyl-CoA hydratase in *B. ubonensis* (ZP_02380855.1), elongation factor 3 in *P. tritici-repentis* (EDU48512.1), regulatory protein Ada in *A. thermophila* (BAJ64916.1), transcriptional regulator in *Micromonospora sp*. (EEP74779.1), PadR-like family transcriptional regulator in *Opitutus terrae* (ACB77801.1), winged helix family two component transcriptional regulator in *B. sp.* (ADX58638.1), GTP-binding protein TypA in *W. chondrophila* (ADI37545.1).

Previously, Loch et al [24] reported that UL49.5 Δ23–32 neither inhibited nor degraded the TAP. Additionally, they reported that like the deletion of entire UL49.5 CT, deletion of two residues 95RG96 at the C terminus failed to induce proteosomal degradation of the TAP complex. Based on a conserved RXE motif in the corresponding U_L_49.5/gN regions of BHV-1, EHV-1 and PRV, they predicted that the RXE motif might be important for these functions. Our results proved that the conserved RXE motif is indeed important for BHV-1 U_L_49.5 TAP inhibition and TAP degradation functions. Previous studies predicted that wt U_L_49.5 binds to TAP complex through its transmembrane domain, and that the binding of wt U_L_49.5 leads to conformational arrest of TAP complex [24]. Subsequently, the luminal domain RXE motif signaling to the C-terminal residues 95RG96 across the ER membrane is necessary for triggering the degradation of the TAP complex. In this study, our results demonstrated that in the absence of either U_L_49.5 luminal domain residues RXE or CT residues, TAP is not degraded. Therefore, both the sequence elements are equally important for the U_L_49.5 mediated TAP degradation. Based on our results on TAP inhibition, it is also reasonable to predict that both the RRE motif and the CT may communicate in a similar fashion, noted above, to cause efficient U_L_49.5 mediated TAP inhibition synergistically. However, they are not equally important for TAP inhibition because deletion of RXE motif had significantly higher TAP inhibition compared to CT-null. Interestingly enough, a BLAST search (program BLASTP 2.2.25+) of NCBI protein data bank revealed that similar RRE/RXE motif is highly conserved in a number of bacterial proteins important for ATP dependent enzymatic activity, transcriptional regulator, GTP and ATP binding proteins (Fig. 9). Currently, it is not known whether the highly conserved RXE motifs in any of the proteins listed in Fig. 9 actually binds to ATP or are required for their ATP mediated functions. Recently, it was reported that BHV-1 U_L_49.5 does not interfere with binding of ATP to TAP. However, EHV-1 U_L_49.5 containing similar RXE motif inhibits TAP by interfering with ATP binding to TAP. It remains to be seen whether the RXE motif in EHV-1 U_L_49.5 binds to ATP directly or interacts indirectly to prevent ATP binding to TAP. Loch et al [24] suggested that BHV-1 U_L_49.5 inhibits TAP primarily by degrading TAP. While that might the case for the wt U_L_49.5 where TAP is degraded, retention of significant TAP inhibition property of U_L_49.5 lacking the CT where TAP is not degraded, requires an alternative mechanism of TAP inhibition. Taken together, our results in this study and since the motif is highly conserved in the enzymatically active proteins requiring ATP or in ATP binding proteins, we predict that in the absence of CT residues, U_L_49.5 luminal domain RRE motif may interact with or compete in some fashion for ATP binding to TAP and blocks the peptide transport. This needs to be tested in future studies.

Both in BHV-1 and EHV-1, U_L_49.5 (gN homolog) is not glycosylated but UL49.5/gM interaction is required for gM processing in the Golgi [21], [22], [23]. In PRV, gN is glycosylated but is not required for gM processing [20]. The analyses of mutant U_L_49.5 proteins' interaction with gM and their effect on gM processing showed that only the mutant U_L_49.5 Δ37–40 CT-null had slightly defective gM processing and mutant UL49.5/gM interaction. Recently, we have determined that alanine exchange mutation of BHV-1 U_L_49.5 residue C42A affected mutant U_L_49.5 interaction with the gM and gM processing in the Golgi but not the TAP inhibition function of U_L_49.5 (Wei and Chowdhury, manuscript in preparation). Since U_L_49.5 CT-null mediated TAP inhibition function was not affected due to the additional deletion of residues 37–40 and because residue C42 is important for gM binding and not for TAP inhibition (Wei and Chowdhury, manuscript in preparation), we conclude that U_L_49.5 TAP inhibition and gM interaction domains are different and do not overlap.

Since the mutant U_L_49.5Δ30–32 CT-null interaction with the gM and gM maturation was unaffected, and the mutant U_L_49.5 was incorporated in the virion envelope, we interpreted that mutant U_L_49.5 protein conformation was not affected. Results from a recent calf experiment indicated that immunogenicity of and cellular immunity against BHV-1 U_L_49.5Δ30–32 CT-null mutant virus was enhanced when compared with wt BHV-1 (Wei and Chowdhury, in submission). Therefore, by incorporating these U_L_49.5 mutations in a future BHV-1 marker vaccine we could improve the vaccine efficacy of the current BHV-1 gE-deleted marker vaccine.

## Supporting Information

Figure S1 **DNA Sequences of wild type BHV-1 U_L_49.5.** To generate BHV-1 gN CT-null, the nucleotides 238 to 244 were deleted and the nucleotides 246 and 247 are mutated to AA to introduce a strong stop codon TAA.
(TIF)

Figure S2 **Immunoblotting analysis of MDBK U_L_49.5+ cells expressing BHV-1 U_L_49.5.** As a control, mock and BHV-1 wt-infected MDBK cell lysates were immunoblotted with rabbit anti BHV-1 U_L_49.5-specific antibody (1∶400).
(TIF)
