# Supplementary figures and images for: Bovine Herpesvirus Type 1 (BHV-1) UL49.5 Luminal Domain Residues 30 to 32 Are Critical for MHC-I Down-Regulation in Virus-Infected Cells
Source: PLoS One. 2011 Oct 26;6(10):e25742. doi: 10.1371/journal.pone.0025742 (PMC3202525; doi:10.1371/journal.pone.0025742)

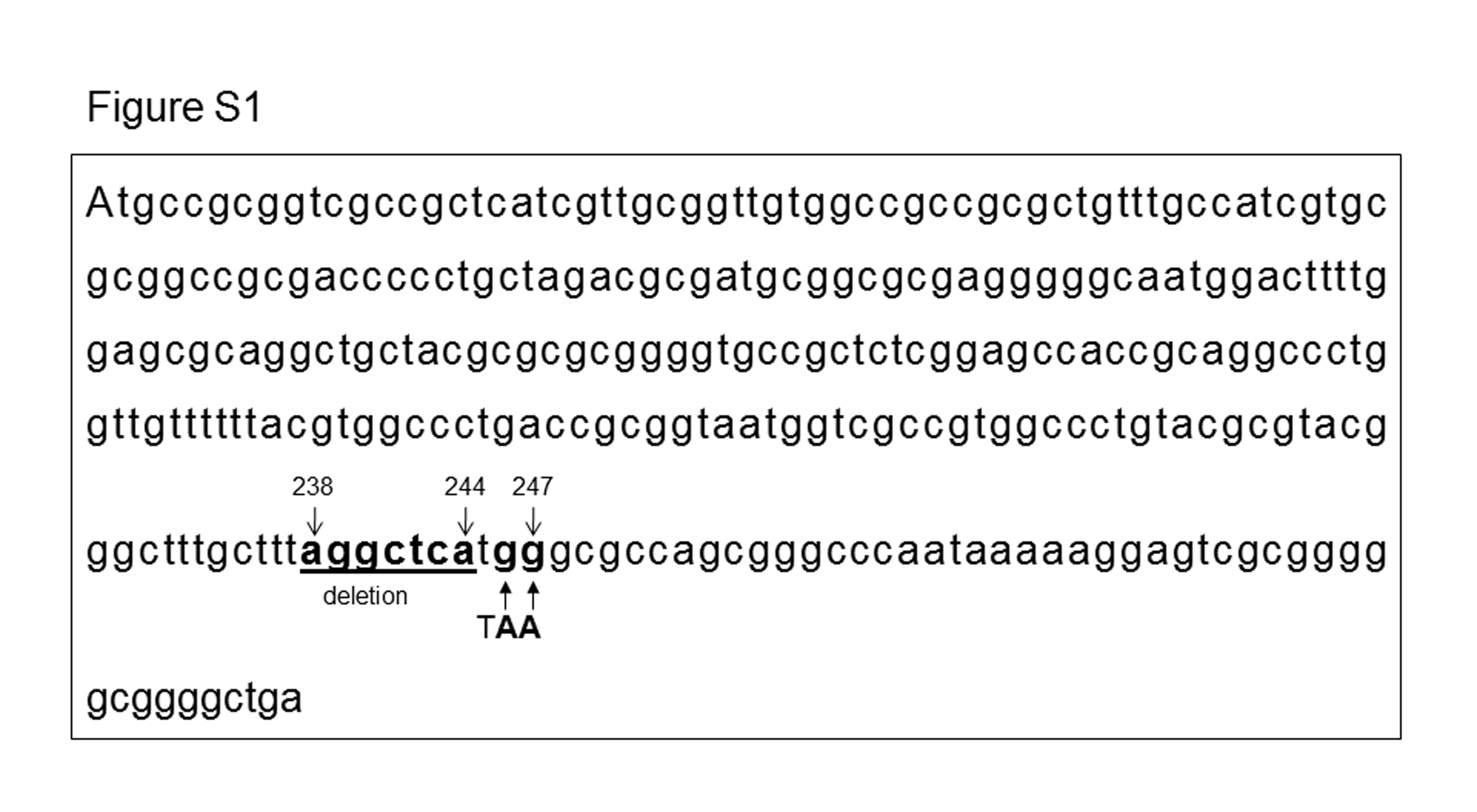

Supplement: Figure S1 — DNA Sequences of wild type BHV-1 UL49.5. To generate BHV-1 gN CT-null, the nucleotides 238 to 244 were deleted and the nucleotides 246 and 247 are mutated to AA to introduce a strong stop codon TAA. (TIF) [file pone.0025742.s001.tif]

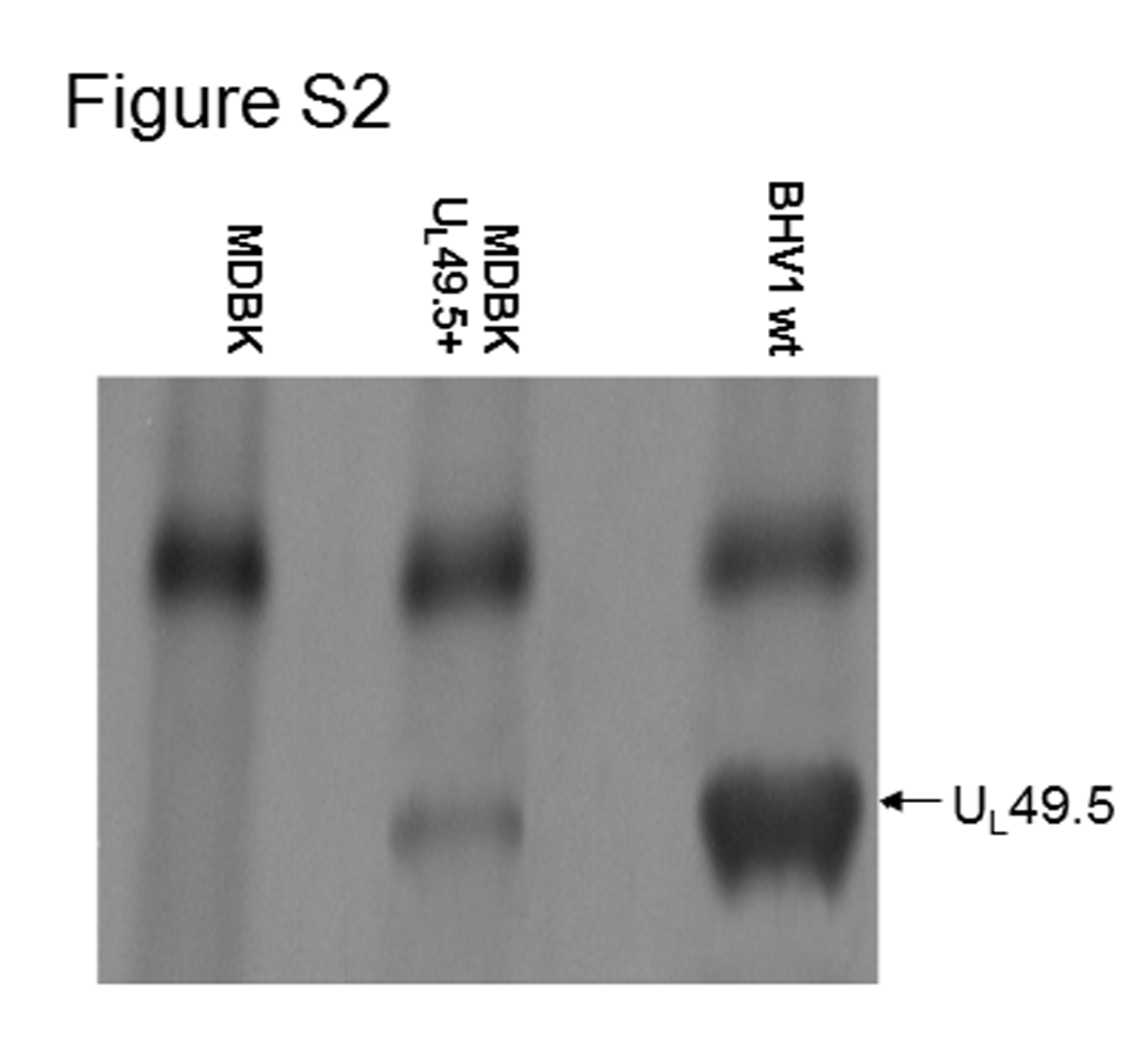

Supplement: Figure S2 — Immunoblotting analysis of MDBK UL49.5+ cells expressing BHV-1 UL49.5. As a control, mock and BHV-1 wt-infected MDBK cell lysates were immunoblotted with rabbit anti BHV-1 UL49.5-specific antibody (1∶400). (TIF) [file pone.0025742.s002.tif]
